# Five cases of rheumatic fever diagnosed after onset of Sydenham chorea

**DOI:** 10.1186/1546-0096-12-S1-P284

**Published:** 2014-09-17

**Authors:** Grazia Cantelmi, Angela Mauro, Antonio Mellos, Carmela Granato, Federica Messa, Maria Francesca Gicchino, Alma Nunzia Olivieri

**Affiliations:** 1 Pediatric, Second University of Naples, Naples, Italy

## Introduction

Acute Rheumatic fever is still a challenge for physicians, it is often not diagnosed at onset and adequately treated.

## Objectives

To describe five cases of Rheumatic Acute fever. Patients came to our department with symptoms of Sydenham's chorea from 2011 to 2014.

## Methods

A. 12y.o., came to our observation for involuntary, arrhythmic movements of the upper limbs. She had a history of pharyngitis with negative throat swab three months before and a 2/6 murmur on cardiac examination. Blood tests showed ESR and TAS increased. On EEG: activity unstable and poorly regulated. On Doppler-echocardiography: mild mitral and moderate aortic regurgitation. Rheumatic Chorea was diagnosed and confirmed by a neuropsychological evaluation. She started penicillin, ASA, and haloperidol therapy. After responding poorly we added Pregabalin, with regression of neurological symptoms. She was on penicillin when Chorea came back nine months after. She is currently on pimozide.

E. 6.5 y.o., admitted to our Department for involuntary movements of the proximal portion of the upper limbs. She had medical history of two pharyngitis treated with antibiotics, in the last year. Performed tests showed high levels of TAS. Rheumatic Chorea was diagnosed. She started penicillin prophylaxis and haloperidol with remission. Three months later there was a relapse. Was added valproate because of an EEG doubt for temporal lobe epilepsy, then responded.

E. 7.7 y.o, had aimless movements of left arm. She was hospitalized for FUO five months before. We did haematological and neuro-radiological examinations. On Doppler-echocardiography: mild aortic and moderate mitral insufficiency. We diagnosed Rheumatic Chorea and Carditis. She started penicillin prophylaxis, valproic acid (8 months) and prednisone (3 months) with disappearance of the symptoms. A year later movements relapsed with extension to left emisoma. She began haloperidol with remission 6 months after.

R. 12 y.o, came to our observation for involuntary movements of limbs and trunk. She had no pathological findings on brain NMR, EEG and echocardiography. Blood tests showed high ESR and TAS value, throat swab positive for GAS. She started penicillin and valproic acid. Two months after she had a severe hypotonia of the right arm so began prednisone with remission of the symptomatology. Two years later, because of a relapse she started Clonazepam.

C.10 y.o presented dyskinetic movements of face and limbs. Blood tests and MRI were negative. On Doppler-echocardiography: mitral regurgitation. He started penicillin and haloperidol. Then, because of the onset of psychosis the dosage was increased. Currently He is in remission.

## Results

CS diagnosis relies on clinical criteria. Therapy is not clearly codified.

## Conclusion

CS is a late neurological consequence of Rheumatic Disease. CS incidence is 10-20%, symptoms may last up 2 years, some of them recurring. The therapy is based on pharmacological correction of neurochemical imbalance of the basal ganglia with antipsychotic, antiepileptic drugs and reduction of inflammation of basal ganglia(corticosteroids). In refractory forms Ig vein can be effective. From 2011 to 2014 we diagnosed 5 cases of SC. Has rheumatic disease changed or can we not diagnose it early enough?

## Disclosure of interest

None declared.

